# Multi-class texture analysis in colorectal cancer histology

**DOI:** 10.1038/srep27988

**Published:** 2016-06-16

**Authors:** Jakob Nikolas Kather, Cleo-Aron Weis, Francesco Bianconi, Susanne M. Melchers, Lothar R. Schad, Timo Gaiser, Alexander Marx, Frank Gerrit Zöllner

**Affiliations:** 1 Institute of Pathology, University Medical Center Mannheim, Heidelberg University, Mannheim, Germany; 2 Institute of Computer Assisted Clinical Medicine, Medical Faculty Mannheim, Heidelberg University, Mannheim, Germany; 3 Department of Engineering, University of Perugia, Perugia, Italy; 4 Department of Dermatology, Venereology and Allergology, University Medical Center Mannheim, Heidelberg University, Mannheim, Germany

## Abstract

Automatic recognition of different tissue types in histological images is an essential part in the digital pathology toolbox. Texture analysis is commonly used to address this problem; mainly in the context of estimating the tumour/stroma ratio on histological samples. However, although histological images typically contain more than two tissue types, only few studies have addressed the multi-class problem. For colorectal cancer, one of the most prevalent tumour types, there are in fact no published results on multiclass texture separation. In this paper we present a new dataset of 5,000 histological images of human colorectal cancer including eight different types of tissue. We used this set to assess the classification performance of a wide range of texture descriptors and classifiers. As a result, we found an optimal classification strategy that markedly outperformed traditional methods, improving the state of the art for tumour-stroma separation from 96.9% to 98.6% accuracy and setting a new standard for multiclass tissue separation (87.4% accuracy for eight classes). We make our dataset of histological images publicly available under a Creative Commons license and encourage other researchers to use it as a benchmark for their studies.

Human solid tumours are complex structures that typically contain several distinct tissue types. Apart from clonal tumour cells, they consist of tumour stroma, immune cell infiltration, necrotic areas and islets of remaining non-malignant tissue. These different tissue types can be distinguished by histopathological evaluation of Hematoxylin and Eosin (H&E) stained tissue sections. In colorectal cancer (CRC), one of the most prevalent cancer types, tumour architecture changes during tumour progression^1^ and is related to patient prognosis^2^. Quantifying the tissue composition in CRC is therefore a relevant task in histopathology.

While manual evaluation of histological slides is still indispensable in clinical routine, automated image processing can provide quantitative and high-throughput analysis of the tumour tissue. In principle, automatic separation of tissue types in histological images can be achieved by different supervised machine learning approaches: in *cell morphology based methods*, individual cells are segmented and then classified into different categories such as tumour cells, stroma cells and immune cells. This approach has been successfully used by various groups (see Xu *et al*.^3^ for an overview) and has led to new candidate biomarkers^4^,^5^,^6^. A different type of tissue classification methods is based on *texture*. The term *texture* refers to specific properties of the internal structure of image regions, for example *rough* versus *smooth* or *oriented* versus *randomly dispersed* (among others)^7^,^8^,^9^. In medical image analysis, *texture based methods* are very useful to classify tissue types^10^,^11^. Typically, these methods extract texture features first^8^,^12^,^13^,^14^, then feed the features into a classifier to predict the tissue type^9^,^15^,^16^.

However, when it comes to classifying tissue types in CRC histological images, all published methods invariably show two common limitations: first, they consider only two categories of tissue (tumour and stroma), which makes these approaches unsuitable for more heterogeneous parts of the tumour^8^,^12^; second, all studies used their own image data set which prohibits quantitative comparison of classification performance. Whereas publicly available benchmarking datasets exist for image classification problems such as face recognition^17^, handwriting recognition^18^, universal computer vision problems^19^ and texture classification^20^,^21^, no such data are available for histopathological tissue classification.

The aim of this study is to fill this gap. To this end we assembled, tested and publicly released a comprehensive image set of all relevant types of tissue within colorectal cancer samples. We used the dataset to compare several state of the art texture features and classifiers and to determine which combination is best suited for a multiclass tissue classification problem.

## Material and Methods

### Ethics statement

All experiments were approved by the institutional ethics board (medical ethics board II, University Medical Center Mannheim, Heidelberg University, Germany; approval 2015-868R-MA). The institutional ethics board waived the need for informed consent for this retrospective analysis of anonymized samples. All experiments were carried out in accordance with the approved guidelines and with the Declaration of Helsinki.

### Dataset

**Figure 1:**
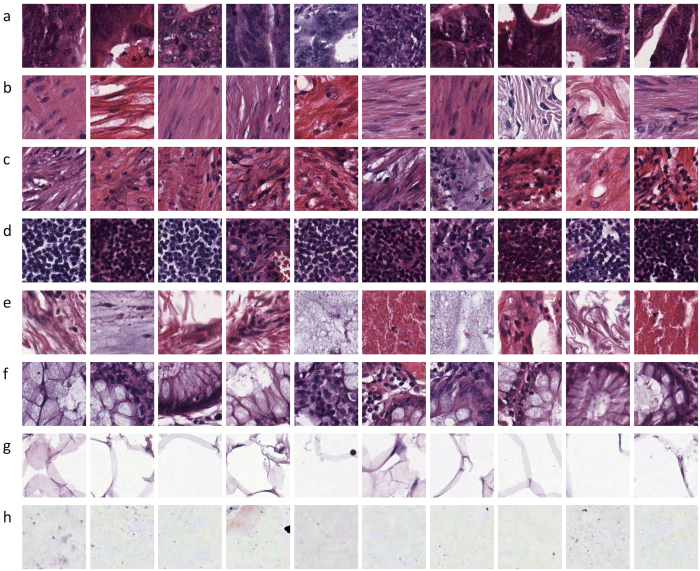
Representative images from our dataset. Here, the first 10 images of every tissue class in our dataset are shown. They represent the wide variation of illumination, stain intensity and tissue textures present in routine histopathological images. Images were extracted from 10 independent samples of colorectal cancer (CRC) primary tumours. (**a**) tumour epithelium, (**b**) simple stroma, (**c**) complex stroma (stroma that contains single tumour cells and/or single immune cells), (**d**) immune cell conglomerates, (**e**) debris and mucus, (**f**) mucosal glands, (**g**) adipose tissue, (**h**) background.

Ten anonymized H&E stained CRC tissue slides were obtained from the pathology archive at the University Medical Center Mannheim (Heidelberg University, Mannheim, Germany). Low-grade and high-grade tumours were included in this set; no further selection was applied. The slides were first digitized as described before^22^. Then, contiguous tissue areas were manually annotated and tessellated, creating 625 non-overlapping tissue tiles of dimension 150 px × 150 px (74 μm × 74 μm). Thus, texture features of different scales were included, ranging from individual cells (approx. 10 μm, e.g. Fig. 1d) to larger structures such as mucosal glands (>50 μm, e.g. Fig. 1f). The following eight types of tissue were selected for analysis:

- Tumour epithelium;
- Simple stroma (homogeneous composition, includes tumour stroma, extra-tumoural stroma and smooth muscle);
- Complex stroma (containing single tumour cells and/or few immune cells);
- Immune cells (including immune-cell conglomerates and sub-mucosal lymphoid follicles);
- Debris (including necrosis, hemorrhage and mucus);
- Normal mucosal glands;
- Adipose tissue;
- Background (no tissue).

Together, the resulting 625 × 8 = 5000 images represented the training and testing set of the classification problem described in the following sections. The first 10 images of each class are shown in Fig. 1. Average staining intensity considerably varied between the tissue samples, reflecting the usual variability in routine histopathological slides. We took care that each of the classes listed above contained both bright and dark samples so that no bias in terms of average greyscale intensity was introduced (Fig. 1). In addition to these images, we also extracted ten larger images of dimension 5000 px × 5000 px from tissue regions different from those used for the smaller images. These ten images constituted an application set and were used to test the different combinations of texture features/classifiers in a realistic setting.

### Data usage statement

We release all raw data under a Creative Commons Attribution 4.0 International License (http://creativecommons.org/licenses/by/4.0/). The data can be accessed via the following DOI: 10.5281/zenodo.53169. All source codes used for this study are available under the MIT license (http://opensource.org/licenses/MIT) and can be accessed via the following DOI: 10.5281/zenodo.53735.

### Texture descriptors

To describe the texture of histological images we considered six distinct sets of descriptors that are detailed in the following sections. All images were preliminarily converted to greyscale before computing the texture features. Yet, in the dataset we provide, images are native red/green/blue (RGB) images so that it can also be used to benchmark colour-based texture classifiers.

### Lower-order and higher-order histogram features

Lower-order statistics can be used to describe texture^23^,^24^. We used the gray level histogram of a given image to construct two simple feature sets: 1) one set containing the mean, variance, skewness, kurtosis and the 5th central moment of the histogram (five features); 2) another set composed of the central moments from 2nd to 11th (ten features). In the remainder we refer to the two sets of features as ‘histogram-lower’ and ‘histogram-higher’, respectively. Note that the latter does not contain the mean therefore it is invariant to changes in the average greyscale intensity of the input image (and is therefore less sensitive to staining differences).

### Local binary patterns (LBP)

The third feature set was based on local binary patterns (LBP)^25^. The Local Binary Patterns (LBP) operator considers the probability of occurrence of all the possible binary patterns that can arise from a neighbourhood of predefined shape and size. In this work we considered a neighbourhood of eight equally-spaced points arranged along a circle of radius 1px. This is usually referred to as the ‘8, 1’ configuration^26^. For each position of the neighbourhood a corresponding binary pattern is obtained by thresholding the intensity values of the eight points on the circle at the value of the central point. In our study, the resulting histogram was reduced to the 38 rotationally-invariant Fourier features proposed by Ahonen *et al*.^27^. Other LBP variants have been used for histological texture analysis in other studies^12^,^13^.

### Gray-level co-occurrence matrix (GLCM)

The fourth feature set was based on GLCM features^9^,^24^. In particular, we used four directions (0°, 45°, 90° and 135°) and five displacement vectors (from 1px to 5px). To make this texture descriptor invariant with respect to rotation, we averaged the GLCMs obtained from all four directions for each displacement vector. From each of the resulting co-occurrence matrices we extracted the following four global statistics: contrast, correlation, energy and homogeneity^9^, thereby obtaining 5 × 4 = 20 features for each input image.

### Gabor filters

The fifth set of features was based on Gabor filtering^28^. We applied a bank of Gabor filters to the greyscale image and computed the mean intensity of the resulting Gabor-transformed magnitude images. In particular, we used six directions (0°, 30°, 60°, 90°, 120° and 150°) and six wavelengths (2, 4, 6, 8, 10 and 12 px/cycle). We chose these particular wavelengths because we subjectively observed that the texture structures of interest in histological images (cells, cell nuclei or collagen fibres) typically ranged between 2 px and 12 px. To make this texture descriptor invariant with respect to rotation, we averaged the results obtained from all Gabor filters with identical wavelength over all orientations, thereby obtaining 6 features for each input image.

### Perception-like features

The sixth set included features based on image perception. These are intrinsically different from most texture features, such as LBP or GLCM based features, which are, by contrast, not easily understandable. Tamura *et al*.^7^ showed that the human visual system discriminates texture through several specific attributes that were later on refined and tested by Bianconi *et al*.^8^. The features used in this study were the following five: coarseness, contrast, directionality, line-likeness and roughness. A detailed description of these features is given by Bianconi *et al*.^8^.

### Combined feature sets

Lastly, we investigated whether discriminatory power of the feature sets could be improved by merging features into a concatenated feature vector. As opposed to the *pure* feature sets described before, we subsequently refer to those as *combined* feature sets. First, we ranked the feature sets based on their classification accuracy as described below (histogram-lower > LBP > histogram-higher > GLCM > Perceptual > Gabor). The procedure for accuracy estimation was based on 10-fold cross validation with full sampling. The subdivision into train and test set was repeated 10 times; in each subdivision 90% of the images of the whole dataset was used to train the classifier and the remaining 10% to test it. Accuracy for each classification round was computed as the ratio between the number of images of the test set correctly classified and the total number of images of the test set. The overall accuracy was estimated as the average over the 10 classification rounds.

Then, we successively added pure feature sets to the combined feature sets: *best2* (histogram-lower and LBP), *best3 (best2* and *histogram-higher*, removing the duplicate features that belonged both to *histogram-low* and *histogram-high*), *best*4 (*best3* and *GLCM*), *best5 (best4* and *Perceptual*) and *all6 (best5* and *Gabor*). The different range of the feature vectors was accounted for by standardizing mean and variance of each column of the feature matrix before SVM classification.

### Classifiers

We used four classification strategies: 1) 1-nearest neighbour, 2) linear SVM, 3) radial-basis function SVM and 4) decision trees. We recall the basics of each classifier in the following sections.

### 1-nearest neighbour

The Euclidean-distance 1-nearest neighbour (1-NN) is a very simple classifier that is independent of tuning parameters, is easy to implement and has a low risk of overfitting^29^. Before training the classifier, the feature vectors were standardized to have equal mean and variance.

### Linear and radial basis function support vector machine

We employed support vector machines (SVM) with one-versus-one class decisions in an error-correcting output code multiclass model (ECOC)^30^,^31^. We compared linear SVM and radial basis function (rbf, Gaussian) SVM. Before training, the feature vectors were automatically normalized to have equal mean and variance.

### Ensemble of decision trees

Finally, we considered an ensemble of decision trees using the RUSboost method. This method is especially suited for data with unequal group sizes. Although this is not the case in our study (the groups are perfectly balanced), we chose RUSboost because it is considered a fast and robust technique^32^.

### Construction of training and testing set

To train the classifiers we used 10-fold cross validation. The 5000-item dataset was randomly subdivided in 10 parts, and 10 rounds of training and testing were performed. For each subdivision a different 10% subset of the dataset was used for testing while the other 90% was used for training. Because the overall number of images was large (5000 images in total) and the group sizes balanced (625 images per set), randomly distributing the images into training and testing set yielded consistent group proportions, even without an explicit stratification approach.

Two types of classification problems were analysed: a multi-class problem (comprising all 5000 images in 8 classes, i.e. the full dataset) and a two-class problem (comprising only 1250 images, i.e. 625 images of “tumour epithelium” and 625 images of “simple stroma”). The two-class problem was addressed because tumour-stroma separation has been addressed by other studies^8^,^12^, and therefore these results could be quantitatively compared to the results of the present study.

### Multi-channel visualization

After training and testing the classifiers we used them to segment an independent set of 10 images (application set, as described above). Each 5000-pixel square image contained regions of different tissue types: identifying these regions is a common problem in digital histopathology. Each input image was subdivided into 10,000 overlapping 150-pixel square tiles and for each tile the texture features were computed and submitted to the classifier.

### Implementation

The approaches described in the preceding sections have been implemented in Matlab® (R2015b, Mathworks, Natick, MA, USA), and the experiments were carried out on a standard computer workstation (2.2 GHz Intel Core i7, 16 GB 156 RAM). In addition to custom routines developed by the authors and Matlab’s built-in functions, we also used publicly available source code from Bianconi *et al*.^8^ and Ahonen *et al*.^27^. The entire code required to reproduce the experiments is freely available to the public (see “Data usage” section).

## Results

### Performance of pure feature sets in a two-class and multiclass problem

We performed 4 × 6 × 2 = 48 supervised image classification experiments to estimate the accuracy of each combination of one of six feature sets, one of four classifiers for either two (tumour-stroma) or eight target categories (multiclass problem).

**Figure 2:**
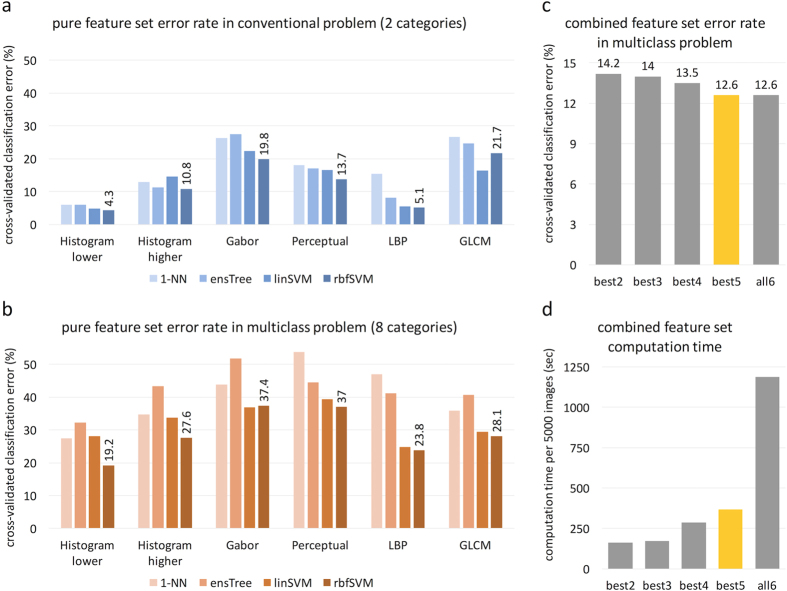
Benchmarking of pure and combined feature sets and four classifiers. (**a**) Experimentally measured error rate in two-category problems (tumour-stroma separation). (**b**) Error rate in multiclass problems (8 tissue categories). Classification accuracy is given as the mean of a 10 experimental runs with 90% of the dataset as training and 10% of the dataset as testing group (10-fold cross-validation). It can be seen that radial basis function (rbf) support vector machine (SVM) outperforms other classifiers, especially in the multiclass setting. (**c**) After testing the pure feature sets, we assessed discriminatory power of the concatenated feature vectors. *Best2* = *histogram-lower* and *LBP*; *best3* = *best2* and *histogram-higher*; *best4* = *best3* and *GLCM*; *best5* = *best4* and *Perceptual*; *all6* includes all features. Accuracy reaches an optimum in the *best5* set (bar highlighted in yellow). (**d**) Computational performance is acceptable in *best5* set but not in the *all6* set. Abbreviations: LBP = local binary patterns, GLCM = gray-level co-occurrence matrix, 1-NN = 1-nearest neighbour, ensTree = ensemble of decision trees, linSVM = linear support vector machine (SVM), rbfSVM = radial basis function SVM.

First, we tested all pure methods, i.e. sets of features obtained with a single texture description method. We found that in a conventional two-class problem, lower order histogram features outperformed the other feature sets (Fig. 2a). Comparing performance of different classifiers with identical feature sets, we found that radial basis function (rbf) support vector machine (SVM) yielded the lowest classification error rate in all but one experiment (Fig. 2a,b).

Specifically, using an rbf SVM in a two-class problem, classification error rate was 4.3% for histogram-lower, followed by 5.1% for LBP. Similarly, in a multiclass problem, histogram-lower and LBP yielded the best results with 19.2% and 23.8% error rates (Fig. 2b).

### Combining feature sets markedly improves classification performance

**Figure 3:**
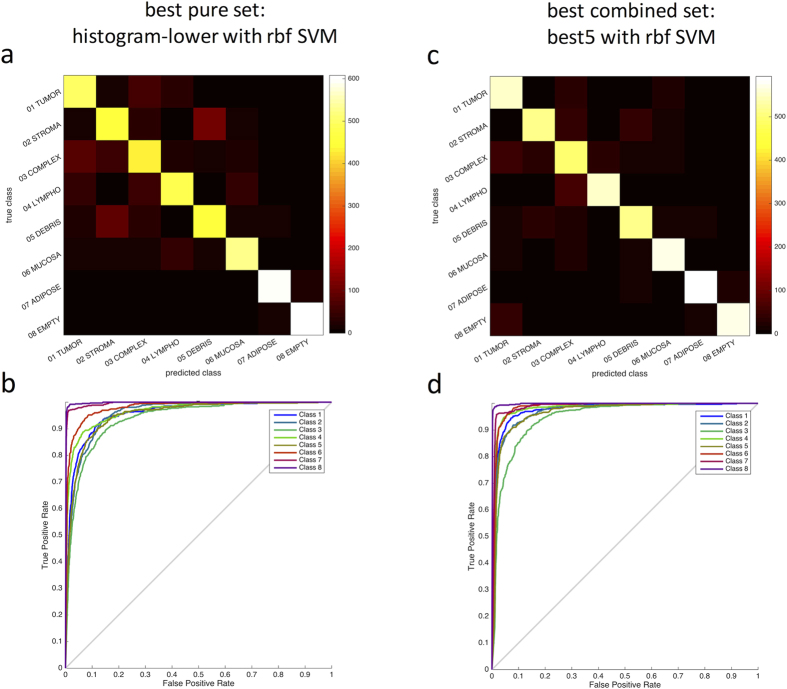
Performance of two classification methods for eight tissue categories. (**a**) Confusion matrix for the best pure feature set *histogram-lower*, classified with a radial basis function (rbf) support vector machine (SVM), (**b**) corresponding receiver operating characteristic (ROC) curves (one-against-all approach), mean area under the curve (AUC) = 0.968. (**c**) Confusion matrix for the best combined feature set (*best5*); (**d**) corresponding ROC with mean AUC = 0.976. The confusion matrices show that *histogram-lower* misclassifies only few samples, but *best5* improves this performance even more. Classes: 1 = Tumour epithelium, 2 = Stroma (simple), 3 = Stroma (complex), 4 = Immune cell conglomerates, 5 = Debris and mucus, 6 = Glands, 7 = Adipose, 8 = Background.

Because the different pure feature sets are conceptually different and measure different aspects of texture, we investigated whether performance could be improved by merging these sets. We ranked the feature sets based on their performance in a multiclass problem and tested five combined sets. This approach markedly improved performance in a two-class setting: In a conventional two-class (tumour-stroma) classification problem, the *best2* set already reached an accuracy of 98.3%, which was only slightly increased by considering more features (*best2, best3*: 98.3%; *best4, best5, all6*: 98.6%). To our knowledge, this accuracy is higher than previously reported accuracies for similar problems – see for instance refs 8,12. In a multiclass setting, the optimal performance was achieved by the *best5* feature set (Fig. 2c) and was 87.4%. Also, computational performance was still acceptable in the *best5* feature set (Fig. 2d) as compared to the *all6* feature set. The confusion matrices and receiver operating characteristic (ROC) curves in Fig. 3 show that classification errors are approximately equally distributed among all classes.

### Assessing classification performance in complex images

**Figure 4:**
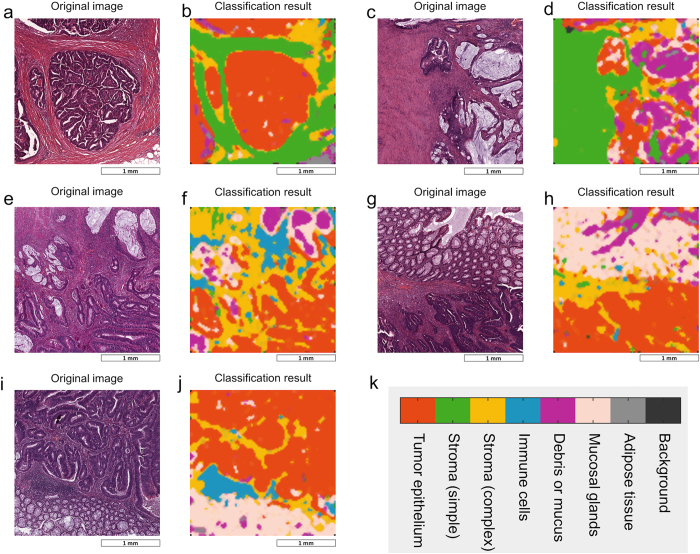
Application of trained classifiers to unknown images. (**a–j**) After training and testing classification performance, we applied the trained classifier to a set of unknown images with highly intermixed textures of which this figure shows 5 representative examples. The left panel (**a,c,e,g,i**) shows the original image and the right panel (**b,d,f,h,j**) shows the classification result in a colour code (observe the legend in (**k**)). Each image was divided into 10,000 patches and each patch was assigned to one of eight tissue classes. For this experiment, we used a combined feature set (*best5*), classifier was a 10-fold cross validated radial basis function (rbf) support vector machine (SVM) with 1-vs-1 class decisions in an error-correcting output code multiclass model (ECOC). Colour-coded classification maps were smoothed by a 3 × 3 median filter and enlarged with bicubic interpolation.

**Figure 5:**
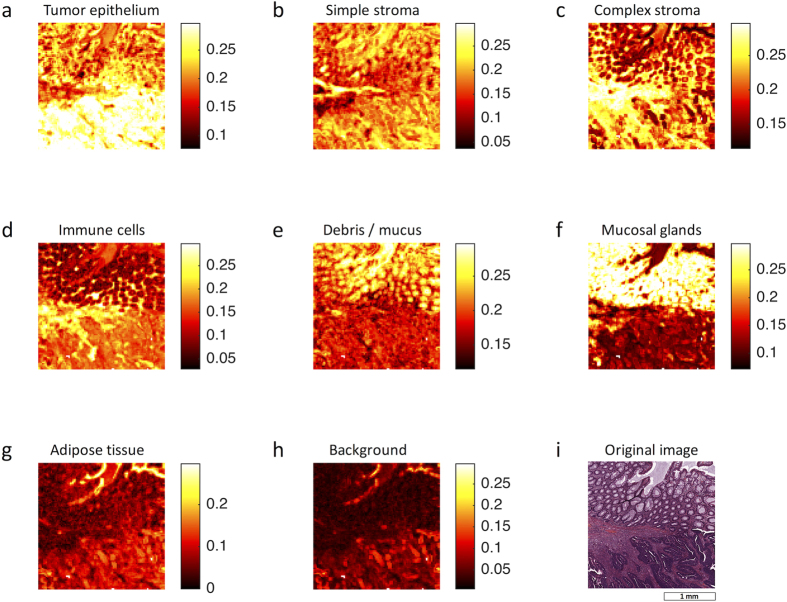
Class probabilities in an example image. For each of 10,000 image patches, the probability of belonging to any of eight tissue classes is shown for an example image. These probability maps correspond to the “committee votes” that a given classifier casts for each image block. (**a–h**) Probability maps, (**i**) original image.

**Figure 6:**
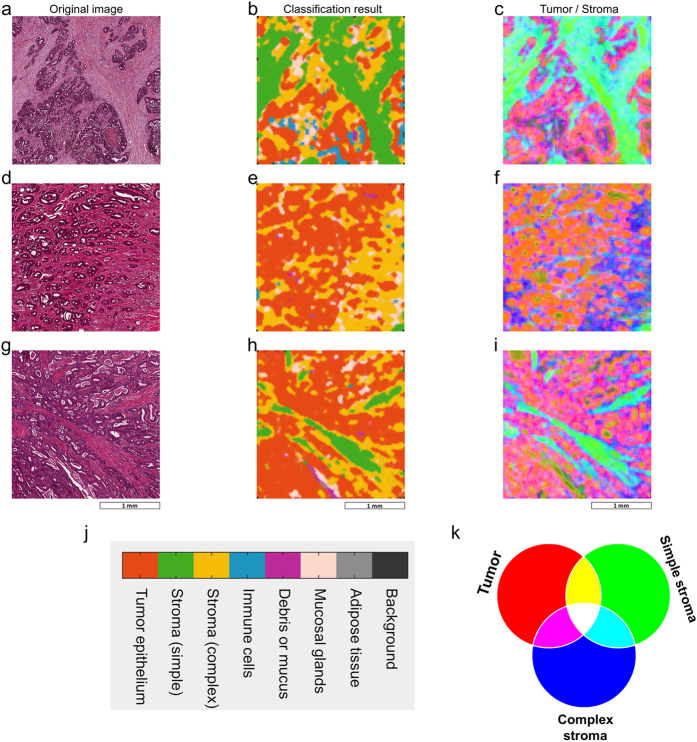
Alternative visualization of classification results. (**a,d,g**) Original image, (**b,e,h**) classification decision, (**c,f,i**) committee votes. The committee votes for tumour, simple and complex stroma in each image block are shown as a false colour visualization with red = tumour, green = simple stroma, blue = complex stroma. This visualization shows spatial overlap of textures. For some tissue regions, this visualization can clarify classification results better than just showing a single classification decision. For this figure, we used images from the application set that primarily consisted of tumour and stroma. (**j**) Legend to (**b**,**e**,**h**). (**k**) Legend to (**c**,**f**,**i**).

To subjectively assess classification performance, we used the best performing classification method (*best5* feature set and rbf SVM) with our application set. This set consists of 10 images that were independent of the training/testing data and contained difficult, intermixed textures. Qualitatively, the resulting segmentation (Fig. 4) shows good separation among the eight tissue classes. Furthermore, the probability maps (Fig. 5) confirm that the class distribution correlates well with the subjective evaluation of the original image (Fig. 5i). To better visualize the classification of mixed tissue types, we also provide a false-colour representation of tumour-stroma separation in Fig. 6. As can be seen, simple stroma and complex stroma gradually fade into each other, and complex stroma tends to cluster in the proximity of tumour epithelium.

### Correlation analysis confirms usefulness of feature combination

**Figure 7:**
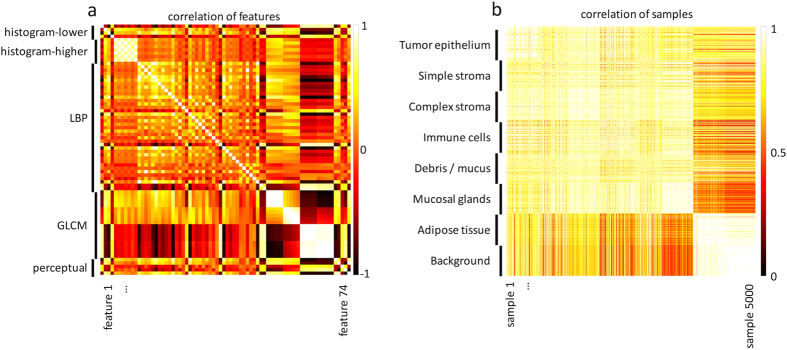
Correlation analysis of the final feature vector. (**a**) Correlation (Pearson’s correlation coefficient) of all 74 features in the *best5* feature set. It can be seen that some subsets of *best5* are highly internally correlated (*histogram-higher* and parts of *GLCM*) but that between the feature subsets, very little correlation is observed. (**b**) Correlation of all 5000 samples in the image dataset as measures by the correlation of their 74-dimensional feature vector. Already in this very simple non-parametric analysis, the individual tissue groups form visually discernible clusters.

In our study, the best classification performance was achieved by the *best5* set, a combined feature set comprising histogram-based features as well as GLCM, LBP and perceptual features. We performed a correlation analysis of the concatenated 74 dimensional feature vectors and found that there was little correlation between the feature subsets (Fig. 7a). This indicates that the feature sets measure different aspects of texture and shows that combining *pure* feature sets may indeed be useful. A correlation analysis of all 5,000 feature vectors (one vector for every image) showed that images of a given class form distinct clusters (Fig. 7b).

## Discussion

### Major findings

In this paper we investigated the use of texture analysis for discriminating between eight different tissue types in colorectal cancer. We found that global lower-order texture measures (“histogram lower”) and the local texture measures GLCM and LBP were able to differentiate multiple tissue types in histological images of colorectal cancer (Fig. 2b) and that a combined approach was particularly effective (Figs 2c and 3c). Another analysis showed relatively little mutual correlation of the individual feature sets (Fig. 7a), thus supporting our approach to combine these different feature sets.

### Texture measures and texture perception

Conceptually, there are many different approaches to measure texture (see Xie *et al*.^33^ and Beyerer *et al*.^24^ for an overview). Histogram-based features are first-order statistics describing the distribution of intensity values in an image. They measure the degree of dispersion of the grey values, the presence/absence of outliers and other properties which reflect the overall structure of the texture. Local texture descriptors such as GLCM^9^ or LBP^26^,^27^ are second-order statistics which consider the joint variability of the grey levels of pairs or groups of pixels. They are among the most used texture descriptors and proved effective in a wide range of applications^34^,^35^. Texture features mimicking the human perception at an abstract level have also been proposed in the literature^7^,^8^ and we also included these methods in our quantitative comparison experiments. Finally, we also tested Gabor filters, another common texture measure based on the response of a set of orientation- and frequency-selective filters^28^,^36^.

### Multiparametric texture visualization

In our study, we became aware of a visualization problem of multiclass texture analysis that, as far as we know, has not been systematically addressed before: Multiclass texture analysis returns multidimensional parametric maps (one probability map for each tissue category). As previous studies mostly addressed two texture types, visualization of these textures was possible by a one-dimensional colour scale^12^. In a previous study, we investigated the use of two-dimensional colour scales to visualize histological imaging data^37^. However, in the present study, we generated an eight-dimensional dataset that cannot be visualized by a low dimensional colour scale. Thus, we implemented and applied three different visualization methods in this study (Figs 4, 5, 6). An alternative would be visualization as an interactive stack of channels.

### Comparison to previous methods

Multi-class texture analysis has not been investigated in CRC histology yet, therefore direct comparison with other studies of the same type is not possible. Similar problems have however been addressed in closely related areas, such as prostate cancer histology. In the case of prostate cancer the neoplastic tissue has a different appearance which is usually classified through Gleason’s grades^38^. There have been structured efforts to automatically classify these grades with reported overall classification accuracies ranging from 74% to 97% (between 4 and 7 tissue categories)^39^,^40^,^41^. A similar approach applied to ovarian cancer histology has been reported to achieve 71.5% accuracy in distinguishing tumour epithelium from different stromal compartments^42^. Lastly, another method applied to breast cancer samples has been reported to achieve 89% accuracy for three tissue categories^43^.

If we compare our results with those just mentioned, we see that the accuracy achieved by our multi-class texture analysis approach is in the same range as was obtained in the other studies. Quite unfortunately, however, the results available in the literature are hard to reproduce and difficult to compare to each other owing to the fact that a) each study uses its own dataset, and b) the datasets are usually not available to the public for further evaluations and comparisons. For this reason, it is not possible to quantitatively compare classification performance of these methods^39^,^40^,^41^,^43^ to our method.

Herein we presented an annotated dataset of 5,000 histological image patches along with a new state of the art classifier for two-class tissue separation and a new method for multiclass tissue separation. By publicly releasing our class-balanced database of histological CRC images we aimed at filling this gap in order to allow histological texture classifiers to be benchmarked on a standard and open-access dataset of colorectal cancer samples.

### Outlook

Whenever a pathologist evaluates a histological image, he or she mentally classifies tissue regions into categories such as “tumour epithelium”, “stroma”, “necrosis”, etc. The method we present in this paper can automate this task. Thus, histological images of colorectal carcinoma can be assessed in a reproducible and high-throughput manner. Automatic analysis is particularly useful when it comes to quantifying the extent of tissue regions. For example, the “tumour-stroma-ratio” (area covered by tumour epithelium divided by area covered by stroma) proved to be an important prognostic factor in a number of neoplastic disorders^2^,^44^,^45^,^46^,^47^. Likewise, the invasion depth of CRC carries profound consequences for the affected patients, but may be difficult to assess in some cases (e.g. when single tumour glands invade much deeper than the tumour bulk). Invasion depth could be automatically quantified by multiclass texture analysis. Another application could be automatic tumour grading, i.e. classification of tumour architecture into G1, G2 or G3. Today, this task is typically done manually (and therefore not always reproducible). Also, multiclass texture analysis could be applied to immunostained images in order to classify distribution patterns of a specific antigen. Another possible application of multiclass texture analysis would be to characterize the morphology of the invasive tumour margin that has been shown to be a powerful prognostic factor for patient survival^48^.

In addition to these technical advances, our texture analysis approach could be used to investigate biological hypotheses based on tissue morphology. For example, stroma tissue has a very heterogeneous morphology (as can be seen in Fig. 1b,c). There is no clear morphological definition of different stroma subtypes (e.g. normal stroma vs. tumour stroma). Multiclass texture analysis could be used to identify morphologically consistent stroma subtypes and investigate biological implications of these subtypes (potentially leading to new morphological biomarkers).

## Additional Information

**How to cite this article**: Kather, J. N. *et al*. Multi-class texture analysis in colorectal cancer histology. *Sci. Rep.* **6**, 27988; doi: 10.1038/srep27988 (2016).
